# Fluoroscopy- and Endoscopy-Guided Transoral Sclerotherapy Using Foamed Polidocanol for Oropharyngolaryngeal Venous Malformations in a Hybrid Operation Room: A Case Series

**DOI:** 10.3390/jcm13082369

**Published:** 2024-04-18

**Authors:** Kosuke Ishikawa, Taku Maeda, Emi Funayama, Naoki Murao, Takahiro Miura, Yuki Sasaki, Dongkyung Seo, Shintaro Mitamura, Shunichi Oide, Yuhei Yamamoto, Satoru Sasaki

**Affiliations:** 1 Department of Plastic and Reconstructive Surgery, Faculty of Medicine and Graduate School of Medicine, Hokkaido University, Sapporo 060-8638, Japan; takumaeda1105@yellow.plala.or.jp (T.M.); sasakiyuki1120@huhp.hokudai.ac.jp (Y.S.); p1ely1@huhp.hokudai.ac.jp (D.S.); shintaro.mitamura@huhp.hokudai.ac.jp (S.M.); shunichi.oide@huhp.hokudai.ac.jp (S.O.);; 2 Center for Vascular Anomalies, Department of Plastic and Reconstructive Surgery, Tonan Hospital, Sapporo 060-0004, Japan

**Keywords:** endoscopy, endovascular procedures, fluoroscopy, hemangioma, cavernous, larynx, mouth, pharynx, sclerotherapy, vascular malformations, venous malformations

## Abstract

**Background:** Treatment of oropharyngolaryngeal venous malformations (VMs) remains challenging. This study evaluated the effectiveness and safety of fluoroscopy- and endoscopy-guided transoral sclerotherapy for oropharyngolaryngeal VMs in a hybrid operation room (OR). **Methods:** Patients with oropharyngolaryngeal VMs who underwent transoral sclerotherapy in a hybrid OR were enrolled. **Results:** Fourteen patients (six females, eight males; median age of 26 years; range, 4–71 years) were analyzed. The symptoms observed were breathing difficulties (n = 3), snoring (n = 2), sleep apnea (n = 1), and swallowing difficulties (n = 1). Lesions were extensive in the face and neck (n = 9) and limited in the oropharyngolarynx (n = 5). A permanent tracheostomy was performed on two patients, while a temporary tracheostomy was performed on five patients. The treated regions were the soft palate (n = 8), pharynx (n = 7), base of the tongue (n = 4), and epiglottis (n = 1). The median number of sclerotherapy sessions was 2.5 (range, 1–9). The median follow-up duration was 81 months (range, 6–141). Treatment outcomes were graded as excellent (n = 2), good (n = 7), or fair (n = 5). The post-treatment complication was bleeding (n = 1), resulting in an urgent tracheostomy. **Conclusions:** Fluoroscopy- and endoscopy-guided transoral sclerotherapy in a hybrid OR can be effective and safe for oropharyngolaryngeal VMs.

## 1. Introduction

Hybrid operation rooms (ORs) have been commonly used in fields such as neurovascular and cardiovascular medicine since the 2010s [1,2]. Improvements in image quality and the ability to perform intraoperative three-dimensional computed tomography (3D-CT) are among the advantages provided by a hybrid OR, allowing for safe and minimally invasive interventions. In hybrid ORs, the imaging device is directly connected to the operating table and navigation system, and all devices are directly controlled by the attending surgeon or radiological technologist [3].

Venous malformations (VMs) are the most frequent type of congenital vascular malformation, developing in the head and neck region more often than in the trunk or extremities [4]. Oropharyngolaryngeal VMs often interfere with swallowing and respiration [5,6]. Sclerotherapy has become a common and less invasive therapeutic option for VMs [4]. However, due to their anatomical location, sclerotherapy and the subsequent airway management of oropharyngolaryngeal VMs remains challenging. Therefore, multimodality-guided sclerotherapy is the preferred approach to ensure safety and reproducibility [7,8,9]. Thus, we used a hybrid OR to provide high-resolution DSA and 3D-CT for the sclerotherapy of oropharyngolaryngeal VMs.

This study aims to evaluate the effectiveness and safety of fluoroscopy- and endoscopy-guided transoral sclerotherapy for oropharyngolaryngeal VMs in a hybrid OR. To our knowledge, this is the first study using a hybrid OR for the sclerotherapy of vascular malformations.

## 2. Materials and Methods

### 2.1. The Hybrid Operation Room and the Endoscopy

**Figure 1:**
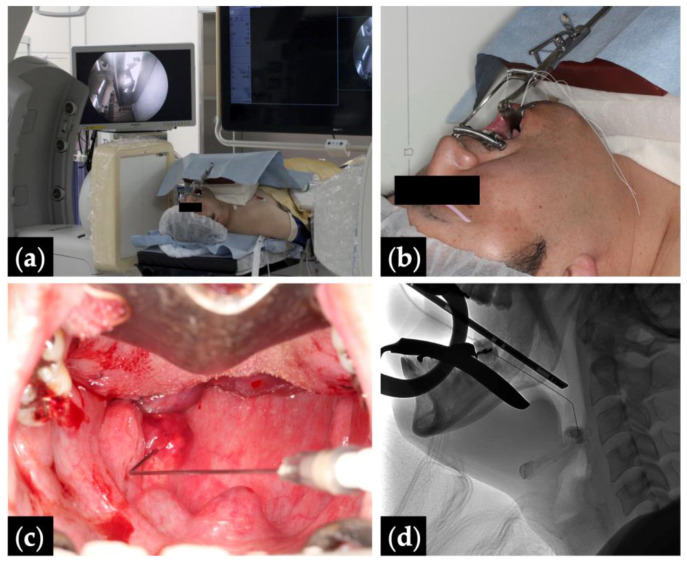
Fluoroscopy- and endoscopy-guided transoral sclerotherapy: (**a**) treatment position with monitors depicting the fluoroscopy (right) and the endoscopy (left); (**b**) Patient 10 positioned head tilt–chin lift with a Dott mouth gag and a tongue blade, and intubated via a tracheostomy; (**c**) endoscopic view with the pharyngeal lesion punctured by an angled angiocatheter; and (**d**) intraoperative fluoroscopic view.

The hybrid OR was installed with the Allura Xper FD20 X-ray system (Phillips, Best, The Netherlands) in combination with the radiolucent carbon-fiber operating table (Maquet Magnus, Phillips) at Tonan Hospital in 2016 (Figure 1). Digital subtraction angiography (DSA) was performed using this system. Endoscopy was performed using rigid telescopes of 0° or 70° connected to a camera head (OTV-S7ProH-HD-12E), a video processor (OTV-S190), a xenon light source (CLV-S190), and a monitor (OEV261H) (Visera Elite system; all from Olympus Medical Systems, Tokyo, Japan).

### 2.2. Patients and Treatment Indications

The electronic medical charts of all patients with VMs treated between 2016 and 2023 at Tonan Hospital were retrospectively reviewed. Patients with oropharyngolaryngeal VMs who underwent transoral sclerotherapy in the hybrid OR with at least 6 months’ follow-up were enrolled in this study. Data were assessed for age at presentation, sex, symptoms, presence or absence of tracheostomy and its duration, and radiological studies during the follow-up period. Data are presented as median (range) or as numbers.

Symptoms included breathing difficulties, snoring, sleep apnea, and swallowing difficulties. Breathing difficulties were defined as any difficulties or discomfort during breathing, and swallowing difficulties were defined as any difficulties or discomfort during the swallowing of food or drink based on the patients’ report. Indications of the treatment were decided on the basis of symptoms or to reduce considerable risk of future airway obstruction due to the enlargement of the lesions for the patients without symptoms [8]. Patients whose main target lesion was the soft palate were treated without a tracheostomy.

Before the sclerotherapy, all patients underwent magnetic resonance imaging (MRI) to assess the distribution of the lesions. Diagnosis of VM was based on their clinical history and findings on physical examination, ultrasonography, and MRI. All lesions included in this study met the MRI criteria for VMs [10,11,12]. Lesions were categorized as limited in the oropharyngolarynx without cutaneous involvement or extensive in the face and neck with cutaneous involvement. The relationship of categorized lesions with the presence of symptoms was assessed using Fisher’s exact test. Statistical significance was set at *p* < 0.05.

### 2.3. Sclerotherapy Procedures

All patients received intravenous hydration before and after sclerotherapy. All sclerotherapy sessions were conducted under general anesthesia by two surgeons (either S.S. or K.I.). An endotracheal tube was inserted through a tracheostomy or nasal route. The patients were positioned head tilt–chin lift with the tongue pulled forward using a silk suture, and a Dott mouth gag and tongue blade were used to keep the mouth open [13]. To visualize needle placement and facilitate direct cannulation of the lesion, the lesion was punctured with a 22-G angiocatheter under fluoroscopic and endoscopic guidance. Nonionic contrast material (iopamidol 300 mgI/mL, Iopamiron 300; Bayer Schering Pharma, Osaka, Japan) was injected under fluoroscopic guidance to confirm the absence of any dangerous venous drainage.

The sclerosant used was 3% polidocanol (Polidocasklerol 3% injection, 30 mg/mL; Kaigen Pharma, Osaka, Japan). The foamed sclerosing solution was obtained by the mixture of 2 mL of 3% polidocanol, 2 mL of contrast material, and 6 mL of atmospheric air (polidocanol, 6 mg/mL) in two syringes attached using a three-way stopcock [14]. The foamed sclerosing solution was subsequently injected into the lesion until adequate filling of the vascular lesion on DSA or upon reaching our maximum dose of the foamed sclerosing solution (1 mL/kg; polidocanol, 6 mg/kg; off-label use) [15,16]. Generally, the maximum dose of polidocanol is 2 mg/kg of body weight for the sclerotherapy of varicose veins [17]. Three-dimensional computed tomography was performed, and the reconstructed images were obtained immediately in the hybrid OR (Supplemental Video S1).

The interval between each sclerotherapy session was scheduled as 3–12 months to allow the swelling to subside and for the clinical status to be accurately assessed. The number of sclerotherapy sessions, doses of foamed sclerosing solution, and post-treatment complications were reviewed. Subsequent swelling of the treated lesions and exacerbations in pain after sclerotherapy were not considered to be complications [18].

### 2.4. Airway Management after Sclerotherapy

After the sclerotherapy, patients with the tracheostomy had their endotracheal tubes replaced with tracheostomy tubes, and patients without a tracheostomy were extubated in the OR, then transferred to the intensive care unit overnight. Methylprednisolone was administered intravenously to reduce swelling of the treated lesions in patients without a tracheostomy. All patients were on intravenous nutrition on the day of the sclerotherapy and started oral intake from a mixer diet the next day onward.

### 2.5. Evaluation of Outcomes

All patients were examined with MRI before the first sclerotherapy session and at least 6 months after the final sclerotherapy session to compare the volume of the targeted lesion on the axial images. The volume reduction rate was defined as multiplication of the longest diameter by the greatest perpendicular diameter of the largest lesion [19,20]. Treatment outcomes were retrospectively graded by two surgeons who had not performed the treatment, using MRI before and after treatments, as follows: excellent (>50% decrease), good (>25% decrease), fair (<25% decrease), and poor (increase in size). Improvement in symptoms was assessed by reviewing patients’ medical charts documented based on the patients’ report at a later follow-up.

## 3. Results

### 3.1. Patients’ Characteristics

Fourteen consecutive patients (six female and eight male) with oropharyngolaryngeal VMs were included in this study. Their median patient age at presentation was 26 years (range, 4–71). Their symptoms were breathing difficulties (n = 3), snoring (n = 2), sleep apnea (n = 1), and swallowing difficulties (n = 1). Lesions were extensive in the face and neck (n = 9) and limited in the oropharyngolarynx (n = 5). Patients with extensive lesions had more symptoms than patients with limited lesions, although this difference was not statistically significant. (56% vs. 20%; *p* = 0.3).

A permanent tracheostomy was performed in two patients at presentation, while a temporary tracheostomy was performed in five patients, which closed spontaneously in four patients after the end of treatment, after the removal of the tracheostomy tube in three patients (duration, 2–28 months), and operatively in one patient (duration, 55 months). Eight patients are receiving ongoing treatment for oropharyngolaryngeal VMs with further sclerotherapy sessions. Table 1 summarizes the clinical characteristics of the patients.

### 3.2. Treatment Outcomes

The treated regions included the soft palate (n = 8), pharynx (n = 7), base of the tongue (n = 4), and epiglottis (n = 1). The median number of sclerotherapy sessions per patient was 2.5 (range, 1–9). The median dose of foamed sclerosing solution per session was 18 mL (range, 3.5–60; polidocanol, 114 mg, range, 21–360). The median follow-up duration after the first sclerotherapy session was 81 months (range, 6–141). Treatment outcomes were graded as excellent (n = 2), good (n = 7), or fair (n = 5). All symptoms were improved at later follow-up.

**Figure 2:**
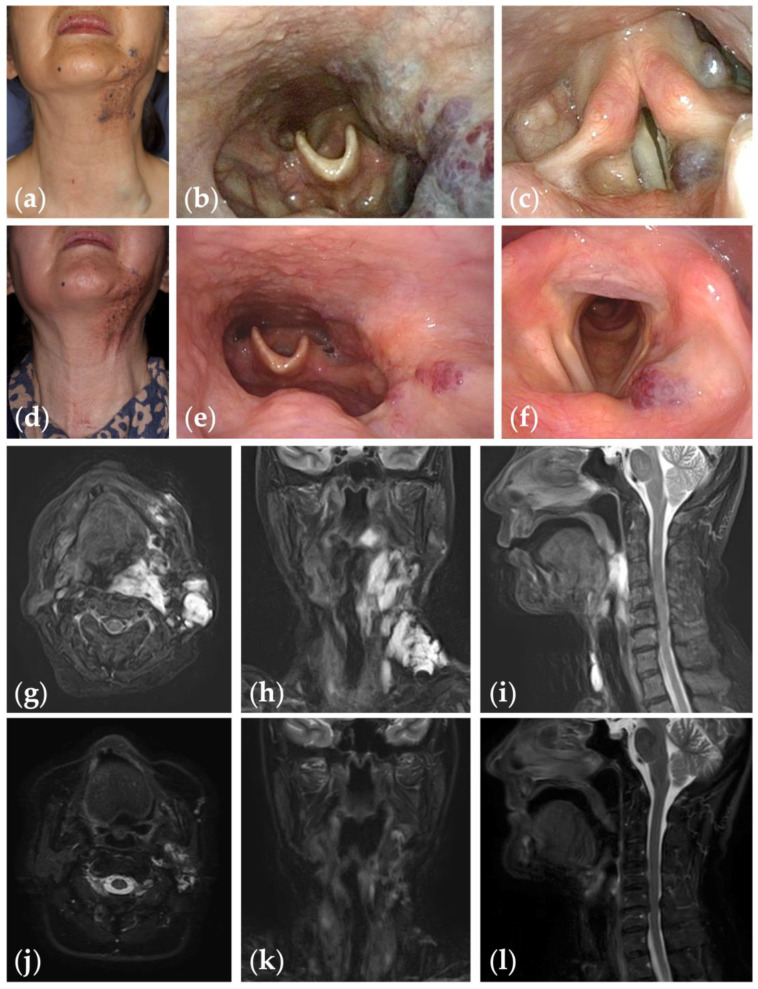
Patient 1 graded “excellent” for treatment outcomes after nine sessions of fluoroscopy- and endoscopy-guided transoral sclerotherapy: (**a**) initial clinical photograph, (**b**) endoscopic photographs of the pharynx; and (**c**) the glottis of a 67-year-old woman with oropharyngolaryngeal venous malformation; (**d**) clinical photograph; (**e**) endoscopic photographs of the pharynx; and (**f**) the glottis six months after the final sclerotherapy session at the age of 75 years; (**g**–**i**) fat-suppressed T2-weighted magnetic resonance images before treatment; (**j**–**l**) short tau inversion recovery T2-weighted magnetic resonance images six months after the final sclerotherapy session.

**Figure 3:**
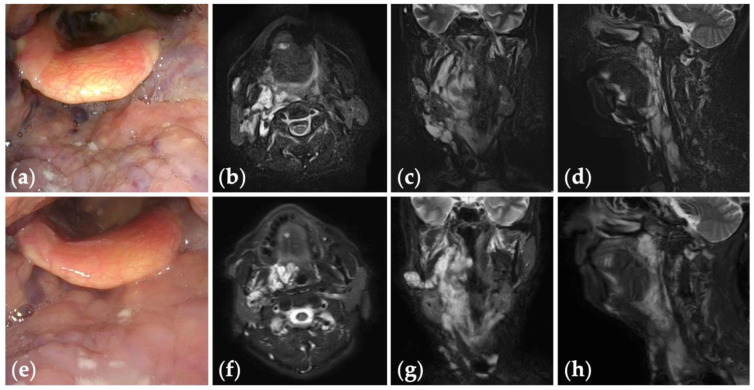
Patient 2 graded “good” for treatment outcomes after seven sessions of fluoroscopy- and endoscopy-guided transoral sclerotherapy: (**a**) an initial endoscopic photograph of the pharynx of a 71-year-old man with oropharyngolaryngeal venous malformation, and (**e**) the latest endoscopic photograph six months after the fifth sclerotherapy session at the age of 73 years; (**b**–**d**) short tau inversion recovery T2-weighted magnetic resonance images before treatment, and (**f**–**h**) three years after the final sclerotherapy session at the age of 78 years.

**Figure 4:**
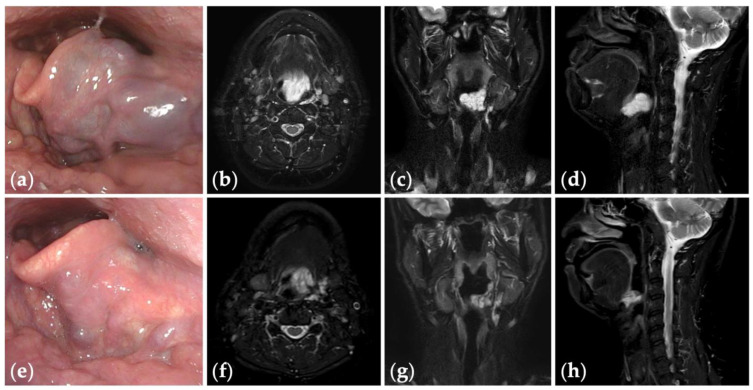
Patient 10 graded “excellent” for treatment outcomes after three sessions of fluoroscopy- and endoscopy-guided transoral sclerotherapy: (**a**) an initial endoscopic photograph of the epiglottis of a 43-year-old man with pharyngeal venous malformation, and (**e**) the latest endoscopic photograph six months after the final sclerotherapy session at the age of 45 years; (**b**–**d**) short tau inversion recovery T2-weighted magnetic resonance images before treatment, and (**f**–**h**) three years after the final sclerotherapy session at the age of 47 years.

The only post-treatment complication was bleeding (n = 1). At the end of the first sclerotherapy session for the lesion of the tongue, persistent oozing bleeding from the base of the tongue was noted. Since packing with gauze did not stop the bleeding, an urgent tracheostomy was performed, and the patient was transferred to the intensive care unit with gauze packing overnight. The next morning, the bleeding had stopped spontaneously. Table 2 summarizes the treatment outcomes of the patients. Representative cases are shown in Figure 2, Figure 3 and Figure 4 (Patients 1, 2, and 10).

## 4. Discussion

Vascular malformations are vascular structural abnormalities that do not present neoplastic proliferation of the vascular endothelial cells, in contrast to hemangiomas [21]. They are classified by the International Society for the Study of Vascular Anomalies (ISSVA) based on their affected vascular components (i.e., capillary, lymphatic, venous, arteriovenous, or combined) [22]. VMs are the most common slow-flow congenital vascular malformations, characterized by dilated venous channels of different sizes and shapes with abnormal smooth muscles [4]. Common symptoms of VMs include pain [23], swelling, bleeding, disfigurement, and functional impairment based on the distribution of lesions [24]. In patients with oropharyngolaryngeal VMs, specific symptoms include breathing, speech, and swallowing difficulties [5,6,25]. As VMs enlarge through expansion stimulated by hormonal changes, trauma, infection, or thrombosis [4,26], asymptomatic oropharyngolaryngeal VMs may pose a potential risk of airway obstruction throughout life.

Imaging is critically important in the diagnosis and treatment planning of vascular malformations [12]. Although ultrasound can provide real-time information of vascularity (fast-flow or slow-flow), MRI provides high-contrast resolution and assessment of anatomical structures without radiation exposure [27]. The standard contrast-enhanced MRI protocol includes pre-contrast axial T1-weighted imaging (WI), multiplanar T2WI, and post-gadolinium multiplanar T1WI sequences [27].

MRIs of VMs show multilocular and lobulated masses that are hypointense or isointense to the muscle on T1WI and hyperintense on T2WI [12]. A more heterogenous appearance can be observed within the setting of hemorrhage or thrombosis in the lesions [11]. These lesions frequently infiltrate into adjacent muscles, joints, nerves, ligaments, and organs [28]. VMs can be confirmed with late enhancement (later than 6 sec after arterial enhancement), absence of flow voids, and the presence of dilated venous spaces [29]. Lower signal areas may represent phleboliths on all imaging sequences [11].

Sclerotherapy is an established minimally invasive treatment option for VMs [4], involving direct cannulation of the lesion and injection of sclerosants [30]. The sclerosants damage the vascular endothelial cells, causing thrombosis and subsequent fibrosis [30]. Absolute ethanol [31], polidocanol [32], ethanolamine oleate [33], bleomycin [34], and sodium tetradecyl sulfate [7] are frequently used sclerosants for VMs of the head and neck [35]. Absolute ethanol is the most effective sclerosant; however, it causes potential side effects such as local tissue necrosis and permanent nerve damage [4]. Polidocanol is a non-ionic detergent and less potent sclerosant with fewer side effects than absolute ethanol [36]. Foamed polidocanol can cause more severe damage to the intima of the veins, compared with the liquid form [14]. We previously preferred to use absolute ethanol and foamed polidocanol for the sclerotherapy of VMs [15,16,37,38], while we used only foamed polidocanol for oropharyngolaryngeal VMs.

Due to their anatomical location, sclerotherapy of oropharyngolaryngeal VMs poses formidable challenges in terms of needle access difficulties, the risk of local side effects, and subsequent airway management. Sclerosants cause swelling, with a considerable risk of airway obstruction [8]. A temporary tracheostomy was necessary before sclerotherapy of the pharynx and the base of the tongue for subsequent airway management [8,26]. Multimodality-guided sclerotherapy is the preferred treatment strategy for oropharyngolaryngeal VMs, allowing for optimal exposure of the targeted lesion [7,8,9]. The combination of fluoroscopy and endoscopy can make it safe and reproducible, especially in a hybrid OR providing high-resolution DSA and 3D-CT.

To the best of our knowledge, this is the first study applying a hybrid OR for the sclerotherapy of vascular malformations. DSA was smoothly performed using the imaging system linked to the radiolucent operating table, controlled by a radiological technologist according to the surgeon’s directions. Finally, the distribution of sclerosant mixed with contrast material could be visualized from the 3D-CT imagery obtained in the hybrid OR.

We demonstrated that fluoroscopy- and endoscopy-guided transoral sclerotherapy can be an effective and safe treatment for oropharyngolaryngeal VMs. Treatment outcomes graded as excellent or good were achieved in 64% of patients. Eight patients had undergone transoral sclerotherapy three or fewer times, with treatment outcomes graded as excellent (n = 1), good (n = 4), or fair (n = 3), although half of them were under ongoing treatment. Patients with extensive lesions required more sclerotherapy sessions than patients with limited lesions (mean number of sclerotherapy sessions per patient, 3.9 vs. 2.6 times, respectively), but 67% of patients with extensive lesions had outcomes graded as excellent or good.

The limitations of this study were its small patient population, a wide range of patients’ ages (4–71 years), and the lack of a control group. Further study is necessary to clarify the effectiveness and safety of fluoroscopy- and endoscopy-guided transoral sclerotherapy in hybrid ORs. Radiation exposure during the procedure is also a concern for patients and medical staff.

## 5. Conclusions

Fluoroscopy- and endoscopy-guided transoral sclerotherapy in a hybrid OR can be effective and safe for oropharyngolaryngeal VMs, enabling intraoperative assessment of the distribution of sclerosant.

## Figures and Tables

**Table 1:** Clinical characteristics of the patients.

Patient	Sex	Age, Years	Distribution of Lesions	Symptoms	Tracheostomy (Duration, Months)
1	F	67	Extensive	Swallowing difficulties	Temporary * (55)
2	M	71	Extensive	−	Temporary (89)
3	F	59	Extensive	–	–
4	M	11	Extensive	Snoring	–
5	F	17	Extensive	–	–
6	F	59	Extensive	Breathing difficulties	Temporary * (2)
7	M	35	Extensive	Breathing difficulties, sleep apnea	Permanent
8	M	41	Extensive	Breathing difficulties	Permanent
9	M	8	Extensive	–	–
10	M	43	Limited	–	Temporary * (9)
11	F	4	Limited	–	–
12	M	11	Limited	–	Temporary * (28)
13	M	16	Limited	–	–
14	F	4	Limited	Snoring	–

F, female; M, male; * closed after the end of treatment.

**Table 2:** Treatment outcomes of the patients.

Patient	Treated Regions	Number of Sclerotherapy Treatments *	Follow-Up, Months	Treatment Outcomes	Complications
1	Pharynx	9	92	Excellent	–
2	Soft palate, pharynx	7	83	Good ^†^	–
3	Soft palate, pharynx	2	141	Good	–
4	Soft palate	2	6	Good ^†^	–
5	Pharynx	1	50	Good ^†^	–
6	Tongue	1	36	Good	Bleeding
7	Soft palate, tongue	5	138	Fair ^†^	–
8	Soft palate	2	130	Fair ^†^	–
9	Soft palate, pharynx	6	138	Fair ^†^	–
10	Pharynx	3	43	Excellent	–
11	Soft palate, tongue	3	79	Good ^†^	–
12	Tongue, epiglottis	5	29	Good	–
13	Pharynx	1	97	Fair	–
14	Soft palate	1	16	Fair ^†^	–

* Only includes fluoroscopy- and endoscopy-guided transoral sclerotherapy in the hybrid operation room; ^†^ treatment is ongoing.

## Data Availability

The data supporting this study’s findings are available from the corresponding author upon reasonable request.

## Supplementary Materials

The following supporting information can be downloaded at: https://www.mdpi.com/article/10.3390/jcm13082369/s1: Video S1: Fluoroscopy- and endoscopy-guided transoral sclerotherapy in the hybrid operation room (Patient 10).

## Abbreviations

CT	computed tomography
DSA	digital subtraction angiography
MRI	magnetic resonance imaging
OR	operation room
VM	venous malformation
WI	weighted imaging
